# The long non-coding RNA lncRNA973 is involved in cotton response to salt stress

**DOI:** 10.1186/s12870-019-2088-0

**Published:** 2019-10-30

**Authors:** Xiaopei Zhang, Jie Dong, Fenni Deng, Wei Wang, Yingying Cheng, Lirong Song, Mengjiao Hu, Jian Shen, Qingjiang Xu, Fafu Shen

**Affiliations:** State Key Laboratory of Crop Biology, College of Agronomy, Shandong Agricultural University, NO. 61 Daizong Street, Tai’an, Shandong 271018 People’s Republic of China

**Keywords:** *Gossypium hirsutum*, lncRNA, Salt treatment, Subcellular location, VIGS

## Abstract

**Background:**

Long non-coding (lnc) RNAs are a class of functional RNA molecules greater than 200 nucleotides in length, and lncRNAs play important roles in various biological regulatory processes and response to the biotic and abiotic stresses. LncRNAs associated with salt stress in cotton have been identified through RNA sequencing, but the function of lncRNAs has not been reported. We previously identified salt stress-related lncRNAs in cotton (Gossypium spp.), and discovered the salt-related lncRNA-lncRNA973.

**Results:**

In this study, we identified the expression level, localization, function, and preliminary mechanism of action of lncRNA973. LncRNA973, which was localized in the nucleus, was expressed at a low level under nonstress conditions but can be significantly increased by salt treatments. Here lncRNA973 was transformed into *Arabidopsis* and overexpressed. Along with the increased expression compared with wild type under salt stress conditions in transgenic plants, the seed germination rate, fresh weights and root lengths of the transgenic plants increased. We also knocked down the expression of lncRNA973 using virus-induced gene silencing technology. The lncRNA973 knockdown plants wilted, and the leaves became yellowed and dropped under salt-stress conditions, indicating that the tolerance to salt stress had decreased compared with wild type. LncRNA973 may be involved in the regulation of reactive oxygen species-scavenging genes, transcription factors and genes involved in salt stress-related processes in response to cotton salt stress.

**Conclusions:**

LncRNA973 was localized in the nucleus and its expression was increased by salt treatment. The lncRNA973-overexpression lines had increased salt tolerance compared with the wild type, while the lncRNA973 knockdown plants had reduced salt tolerance. LncRNA973 regulated cotton responses to salt stress by modulating the expression of a series of salt stress-related genes. The data provides a basis for further studies on the mechanisms of lncRNA973-associated responses to salt stress in cotton.

## Background

Long non-coding (lnc) RNAs are a class of RNAs with transcripts greater than 200 nucleotides in length that do not have the capacity to encode proteins [1, 2]. On the basis of their genomic localization with respect to protein-coding genes, lncRNAs can be classified as long intergenic non-coding (linc) RNAs, long non-coding natural antisense transcripts (lncNATs), long intronic non-coding RNAs and overlapping lncRNAs, which partially overlap with protein-coding genes [3]. The majority of lncRNAs are transcribed by RNA polymerase (Pol) II. In addition, plant-specific RNA polymerases, Pol IV and Pol V, also produce lncRNAs [4]. Unlike mRNAs, lncRNA expression levels are very low and, in general, their conservation is poor. RNA is an integral component of chromatin, and many regulatory RNAs function through interactions with chromatin modifiers and remodelers to change the epigenetic states of target genes. Other RNAs act in a variety of ways to regulate the level of gene expression, such as affecting mRNA splicing, editing, stability and translation [5]. The lncRNA mechanisms of action have *cis* and *trans*-associated functions. *Trans*-acting lncRNAs may act as signals, guides or scaffolds for the chromatin to regulate the expression of target genes located in distant chromosomal domains or even in different chromosomes. *Cis*-acting lncRNA is located upstream and downstream of the encoded protein and interacts with *cis*-acting elements of the promoter or co-expressed genes, thereby regulating gene expression at the transcriptional or post-transcriptional level. Plant lncRNAs may play key roles in flowering time, gene silencing, root organogenesis, seedling photomorphogenesis, re-production, and responses to the biotic and abiotic stresses [6–12].

The functional mechanisms of lncRNAs in many plant species are not yet fully understood and only a few lncRNAs have been fully characterized. In *Arabidopsis thaliana*, lncRNAs, such as cold-assisted intronic noncoding RNA (*COLDAIR*) and cold-induced long antisense intragenic RNA (*COOLAIR*), have been demonstrated to mediate chromatin-modifying activities in the transcriptional silencing of *FLC* (*FLOWERING LOCUS C*) during vernalization [13–17]. Additional regulatory functions of some lincRNAs, such as *At4*, *IPS1* (INDUCED BY PHOSPHATE STARVATION1) and Pi-deficiency-induced long-noncoding RNA1 (*PILNCR1*), act as miRNA decoys through a target mimicry mechanism, thus sequestering the miRNAs with regulatory roles away from their intended target genes [18–20]. As an example of transcriptional regulation by a lincRNA, HIDDEN TREASURE 1 (*HID1*) negatively controls the expression of PHYTOCHROME INTERACTING FACTOR 3 (*PIF3*) and regulates seedling photomorphogenesis [11]. In *Arabidopsis*, lncRNA ELF18-INDUCED LONG-NONCODING RNA1 (ELENA1) enhances resistance against *Pseudomonas syringae*, which can dissociate the FIB2/MED19a complex and release *FIB2* from the *PR1* promoter to enhance *PR1* expression [21]. Furthermore, plant lncRNAs may have important biological roles in responses to abiotic stress. For instance, nucleus-localized drought induced lncRNA (*DRIR*) enhances drought and salt-stress tolerance [22].

Cotton (Gossypium spp.) is an economically important fiber crop, which produces a natural renewable fiber for the textile industry, provides edible protein for livestock feed and is a source of oil and biofuel. The upland cotton *Gossypium* (*G.*) *hirsutum*, accounts for > 90% of the annual cotton production worldwide. However, water limitations and the salinization of cotton cultivation areas are challenges to cotton production. High salinity also causes severe limitations to cotton growth and yield by inhibiting leaf expansion, reducing chlorophyll content and increasing hyperionic and hyperosmotic stresses [23]. A number of protein-encoding genes related to salt stress have been reported, such as *SOS1* [24], *SARP1* [25], *NAC* transcription factors [26]. At present, cotton lncRNA research uses RNA sequencing (RNA-seq) and bioinformatics-based identification methods. The Zhang Laboratory identified a large number of lncRNAs related to fiber development in *G. barbadense* and *G. hirsutum* using RNA-seq [12, 27]. Zou et al. used the strand-specific RNA-seq method to identified 5996 lncRNAs from *G. arboreum* fibers and leaves [28]. Lu et al. used RNA-seq to analyze lncRNAs from nine upland cotton samples in three different environments (control, drought stress and re-watering), and an analysis of transcriptome data indicated that lncRNAs XLOC_063105 and XLOC_115463 may regulate adjacent coding genes CotAD_37096 and CotAD_12502, respectively [29]. Zhang et al. identified lncRNAs and performed an expression and functional comparative analysis in *Verticillium dahlia*-resistant island cotton (*G. barbadense* cv. ‘7124’) and susceptible upland cotton (*G. hirsutum* cv. ‘YZ1’) using RNA-seq [30]. Deng et al. identified lncRNAs related to salt stress in *G. hirsutum*, and lncRNA883 was co-expressed with its downstream gene Gh_D03G0339. Additionally lncRNA973 might be a mimic target of miRNA399 [31]. However, there is limited research on the mechanisms of these lncRNAs, and there are no reports on their functions in salt-stressed cotton.

In this study, we identified and characterized the functions of a lncRNA, lncRNA973. The expression of lncRNA973 was increased by salt treatments. An in situ hybridization analysis revealed that lncRNA973 localized mainly to the nucleus. The overexpression of lncRNA973 enhanced tolerance to salt stress, while reducing its expression had the opposite effect. Transcriptome data and a real-time PCR analysis revealed that lncRNA973 modulates the expression levels of genes involved in the stress response, including reactive oxygen species (ROS)-scavenging, Na^+^ and K^+^ transport, and transcription factors, that may collectively contribute to an enhanced salt-stress tolerance.

## Results

### Identification of lncRNA973 in cotton

**Fig. 1:**
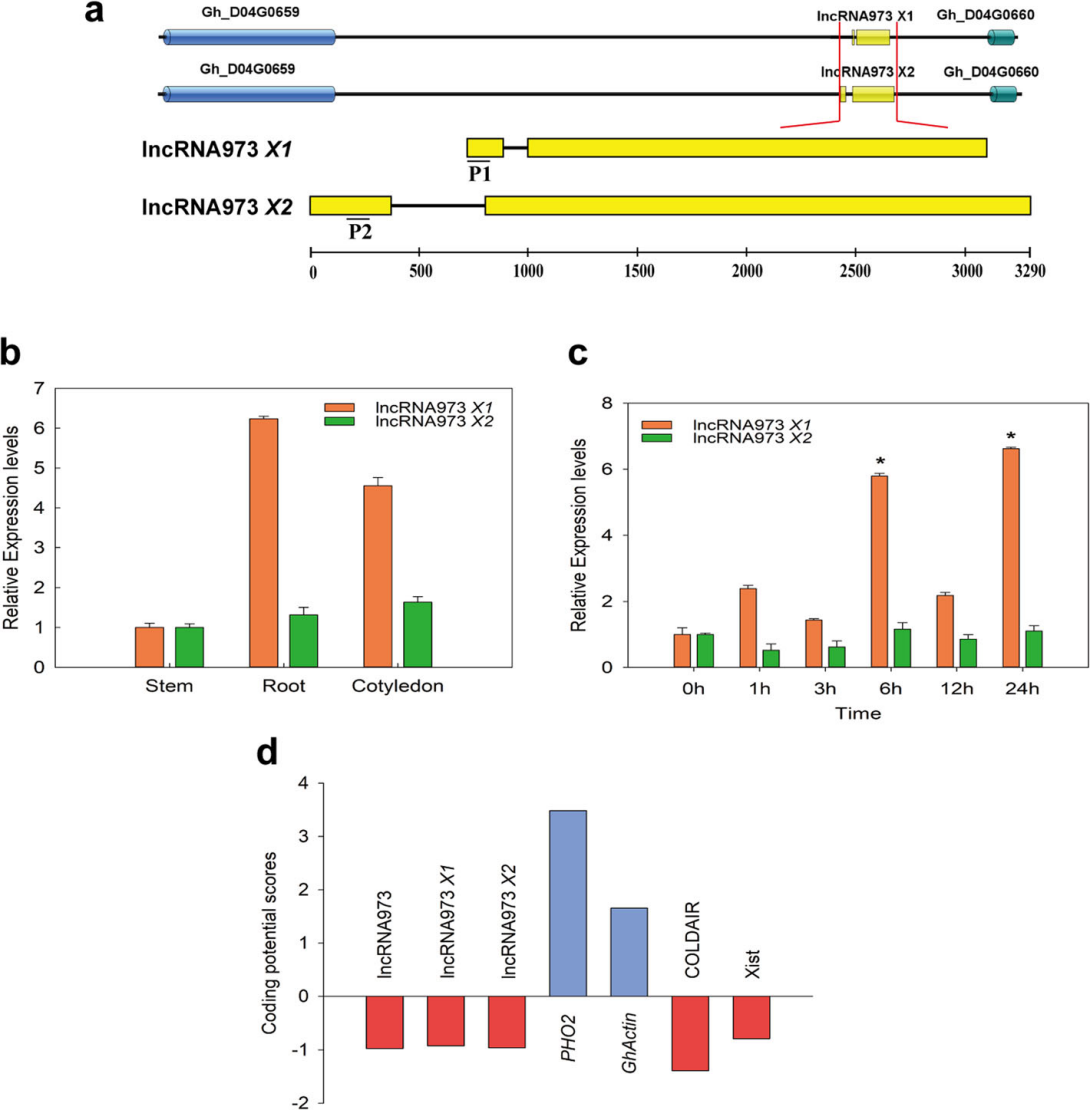
Characteristics of lncRNA973. **a**. Schematic diagram of the location of lncRNA973 in a chromosome. Yellow boxes show the exon of lncRNA973. P1 and P2 indicated the primers used for qRT-PCR to detect the expression level of lncRNA973 *X1* and lncRNA973 *X2*, respectively. **b**. Expression levels of lncRNA973 transcripts in cotton stem, root and cotyledon, determined by qPCR. **c**. Expression levels of lncRNA973 *X1* and *X2* in cotton true leaves treated with 250 mM NaCl at different time points, determined by qPCR. The lncRNA973 expression level was normalized by the *GhUBQ7* expression level. The relative expression levels of lncRNA973 in stem and NaCl-0 h were assigned a value of 1, respectively. Error bars indicate ±SD of three biological replicates, with each measured in triplicate. Samples marked with different letters show a significant difference at *p* < 0.05. **d**. Analysis of the coding potential of lncRNA973. Coding potential scores were generated using the CPC program. *PHO2* and *GhActin* are provided as coding examples, *COLDAIR* and *Xist* represent non-coding examples

Our previous study concerned a salt-related lncRNA, lncRNA973 [31], which was chosen for further analyses. It was identified as a sense intergenic lncRNA, between genes Gh_D04G0659 and Gh_D04G0660. It appears to be transcribed from RNA Pol II, and it contains a polyadenylated 3′ end. We analyzed the structure of lncRNA973, which has introns and exons (Fig. 1a), similar to mRNA. Additionally, lncRNA973 was located on the sense chain of chromosome 4 in the D genome and has two transcripts (lncRNA973 *X1* and lncRNA973 *X2*). The corresponding genomic region was ~ 3.2 Kb, with lncRNA973 *X1* and lncRNA973 *X2* being 2134 bp and 2888 bp in length, respectively. In addition, lncRNA973 *X2* had an exon longer than lncRNA973 *X1*; therefore, the primers for detecting the expression of lncRNA973 *X2* were designed to this exon, primers are designed at this exon location to distinguish them from lncRNA973 *X1*. The expression levels of lncRNA973 *X1* and *X2* in stem, root and cotyledon were analyzed using quantitative real-time PCR (qRT-PCR), and the expression levels of lncRNA973 *X1* in roots and cotyledons were relatively higher, while those of lncRNA973 *X2* were relatively lower in these tissues (Fig. 1b). In addition, we also detected the expression levels of two transcripts of lncRNA973 in cotton true leaves treated with NaCl (250 mmol/L) at different time points (0, 1, 3, 6, 12 and 24 h). The expression of lncRNA 973 *X1* reached its peak at 6 and 24 h, while the expression of lncRNA973 *X2* was low at different time points (Fig. 1c). Thus, we chose lncRNA973 *X1* for further study. In addition, a sequence conservation analysis was performed. Like many other lncRNAs, lncRNA973 does not have homologs in other plants. To investigate the origin of lncRNA973, we analyzed its evolution and homology in other species and found it to be a new gene that principally originated from *G. raimondii*. Its sequence alignment in *G. hirsutum* revealed no homologous regions on chromosome 4 of A genome, which is consistent with its evolutionary origin.

A bioinformatics analysis (http://cpc.cbi.pku.edu.cn/) revealed that lncRNA973 had no coding capacity (Fig. 1d). The ORF Finder online tool was used to predict five ORFs on lncRNA973 *X1* (Additional file 1: Figure S1a), that ranged from 29 to 107 amino acids. Furthermore, according to homology searches of large protein families and domain databases (Pfam and SMART, respectively), no functional domains matching any of the peptides were found.

### Subcellular localization of lncRNA973

**Fig. 2:**
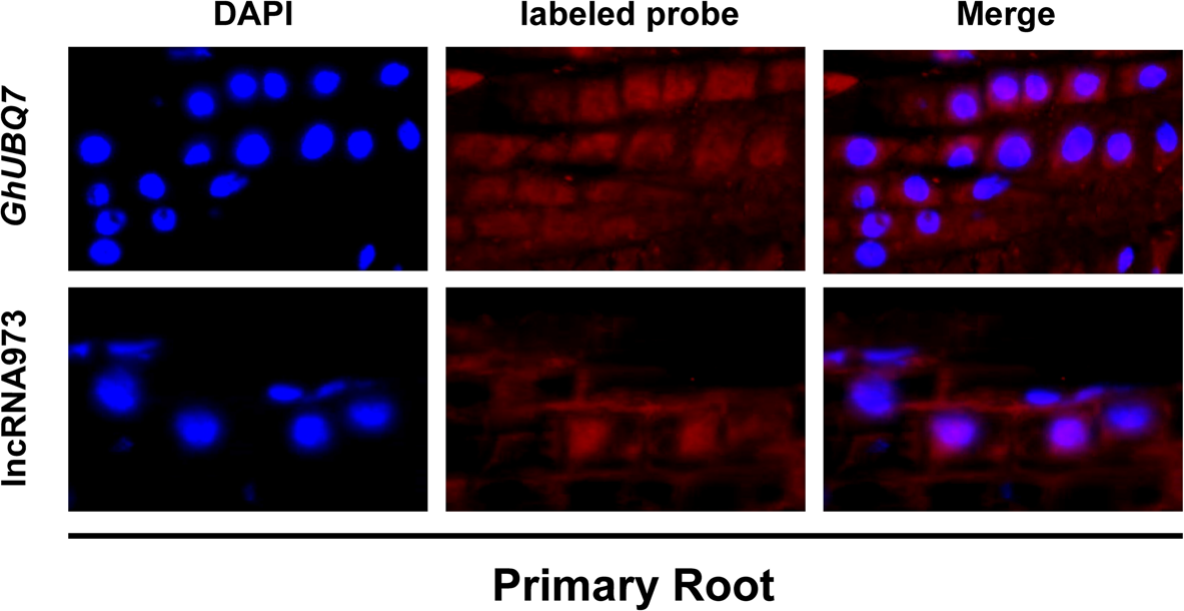
Subcellular localization of lncRNA973. Fluorescence in situ hybridization with DIG-labeled antisense lncRNA973 probes were performed in 10-d-old cotton roots. A housekeeping mRNA (*GhUBQ7*) was used as a positive control. Bar = 20 μm

The function of lncRNAs are associated with their subcellular localization [32]. To investigate the subcellular localization of lncRNA973, fluorescence in situ hybridization in roots was performed using a double-ended DIG-labeled probe specific to lncRNA973. To confirm the subcellular localization of lncRNA973, nuclei were stained with DAPI, and a housekeeping mRNA (*GhUBQ7*) was used as a positive control. For the lncRNA973 and *GhUBQ7* probes, a sequence specificity analysis was performed in the cotton genome, and specific probes were selected for hybridization. The fluorescence signal of lncRNA973 could be seen mainly in nuclei of root cells that were hybridized with the DIG-labeled probe, and was also weak signals in the cytoplasm (Fig. 2). The fluorescence signal of the positive control *GhUBQ7* could be seen in nuclei and cytoplasm. In addition, we also isolated and purified RNA from the cytoplasm and nuclei, respectively, and used for RT-PCR. *U6* was mainly expressed in the nuclei, while tRNA mainly in the cytoplasm. Thus, the separation of nuclei and cytoplasm was distinct. RT-PCR result showed that lncRNA973 was highly enriched in the nuclei (Additional file 1: Figure S1b). These data indicated that lncRNA973 transcripts were localized mainly in nuclei and has a little signal in the cytoplasm.

### Overexpression of lncRNA973 increases salt-stress responses in *Arabidopsis*

**Fig. 3:**
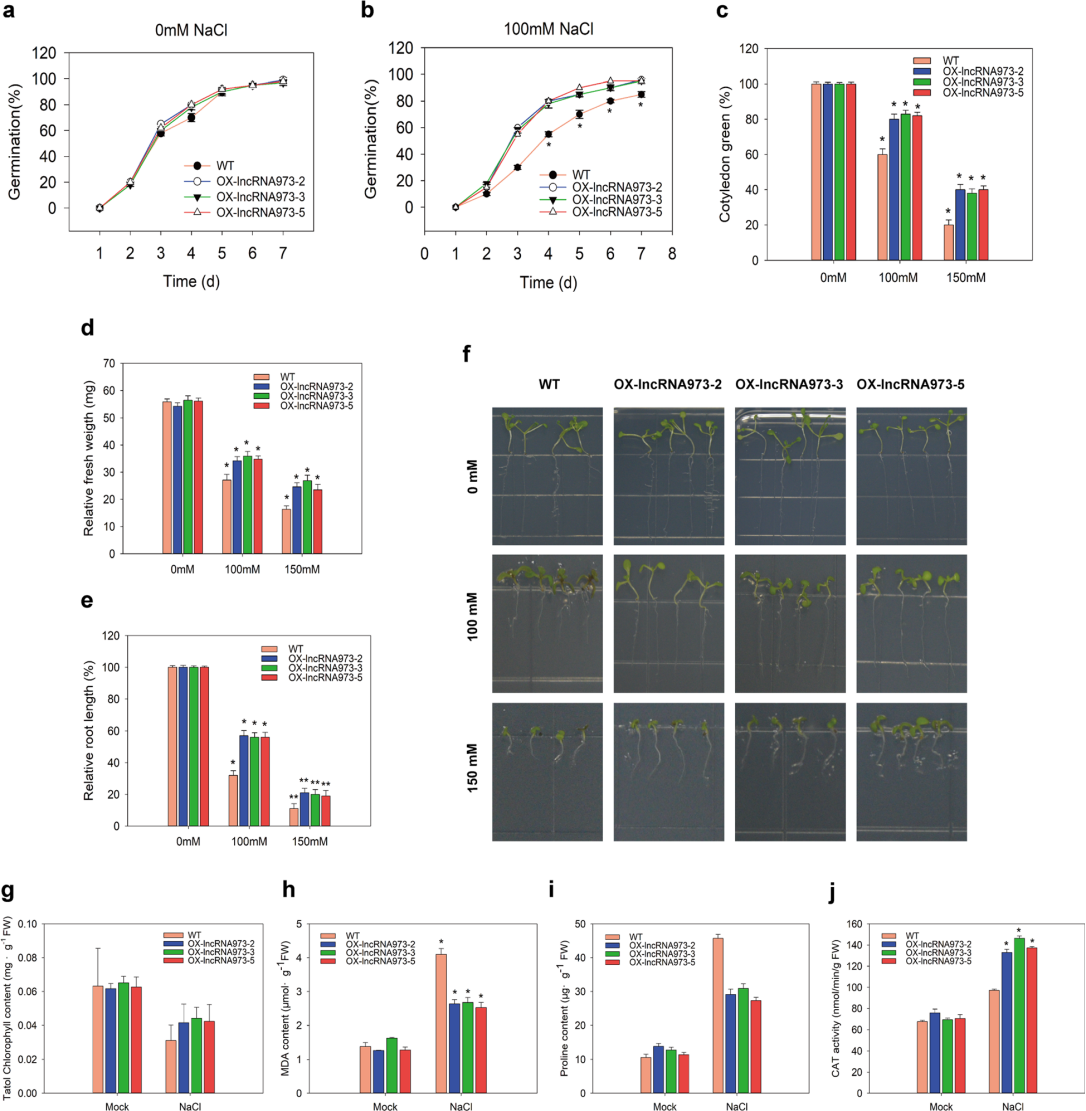
Overexpressing lncRNA973 lines are more tolerant to salt stress. **a**-**b**. Seeds were germinated on ½ MS medium containing 0 and 100 mM NaCl. Germination rates were measured during one week. **c**-**e**. Cotyledon greening, fresh weights and elongation of primary root of 7-d-old seedlings with 0–150 mM NaCl. **f**. Phenotype of primary root growth of 7-d-old seedlings exposed to 0–150 mM NaCl. **g**-**i**. Total chlorophyll, MDA, and Pro contents in the leaves under salt stress. **j**. Activities of catalase (CAT) after treatment with salt and mock. Mock, with water treatment. NaCl: 200 mM NaCl treatment. Vertical bars represent ±SD of the mean with three replicates. Asterisks indicate that mean values are significantly different between the transgenic plants and WT (*p* < 0.05)

To identify the role of lncRNA973 in plant responses to salt stress, the full-length of lncRNA973 transcript was cloned (Additional file 2: Figure S2a), overexpression vector containing the ubiquitin protein promoter *Ubi* was constructed (Additional file 2: Figure S2b), and then *Agrobacterium tumefaciens* (GV3101)-mediated transformation of wild type (WT) *Arabidopsis* (Col-0 ecotype) was performed. Five independent transgenic lines were obtained, and three overexpression (OX-) (OX-lncRNA973–2, OX-lncRNA973–3 and OX-lncRNA973–5) were chosen for further study (Additional file 2: Figure S2c). The transgenic lines were not phenotypically different from the control when untreated. Real-time PCR was performed to check the lncRNA973 transcript expression in transgenic *Arabidopsis*. The expression levels of lncRNA973 in transgenic plants were significantly greater than in WT (Additional file 2: Figure S2d). To investigate the transgenic plants’ abilities to regulate responses to salt stress, wild type (WT) and transgenic *Arabidopsis* seeds (OX-lncRNA973) were placed on solid 1/2 MS medium supplemented with 0 mM, 100 mM and 150 mM NaCl. Then, we determined the germination rates of seeds treated with different salt concentrations from 1 to 7 d and the phenotypes after seed germination. The OX-lncRNA973 lines displayed no abnormal phenotypes under normal (0 mM) conditions. The germination rates of OX-lncRNA973 lines grown under normal (0 mM) conditions resulted in no abnormal phenotypes compared with that of the WT (Fig. 3a). At 3 d after treatment with 100 mM NaCl, the seed germination rates of the OX-lncRNA973 lines were 55 to 60% and are greater than that of the WT. At 7 d salt treatment, the mean seed germination rate of the OX-lncRNA973 lines was ~ 95%, which was higher than that of WT (~ 80%) (Fig. 3b). In addition, the fresh weights, root lengths and cotyledon greening of WT and OX-lncRNA973 lines changed under NaCl-treatment conditions. Exposure to 100–150 mM NaCl also inhibited cotyledon greening in OX-lncRNA973 seedlings, but the effect was less severe than in WT (Fig. 3c). The OX-lncRNA973 seedlings had greater fresh weights than the control (Fig. 3c). The root elongation was significantly inhibited by 100–150 mM NaCl, while the OX-lncRNA973 seedlings had greater tolerance levels to salt stress as indicated by root growth (Fig. 3e-f). Additionally, we examined several physiological indicators to discuss the tolerance of OX-lncRNA973 plants to salinity stress, for example, malondialdehyde (MDA), proline (Pro), chlorophyll content and catalase (CAT) activity. Under normal growth conditions, the contents of chlorophyll, MDA and Pro and the CAT activity in WT and OX-lncRNA973 lines were similar. However, after treatment with 200 mM NaCl, the total chlorophyll content decreased, and the chlorophyll content of WT plants decreased more than the OX-lncRNA973 plants (Fig. 3g). The MDA and proline contents were elevated in both WT and OX-lncRNA973 plants, and WT plants increased more than OX-lncRNA973 plants (Fig. 3h-i). The CAT activity of WT plants was inhibited after treatment with NaCl, and the CAT activity of OX-lncRNA973 plants was higher (Fig. 3j). Therefore, phenotypic analysis and physiological indicators of WT and OX-lncRNA973 plants showed OX-lncRNA973 plants were more tolerant to salt stress than control plants.

### Silencing lncRNA973 leads to susceptibility to salt stress

**Fig. 4:**
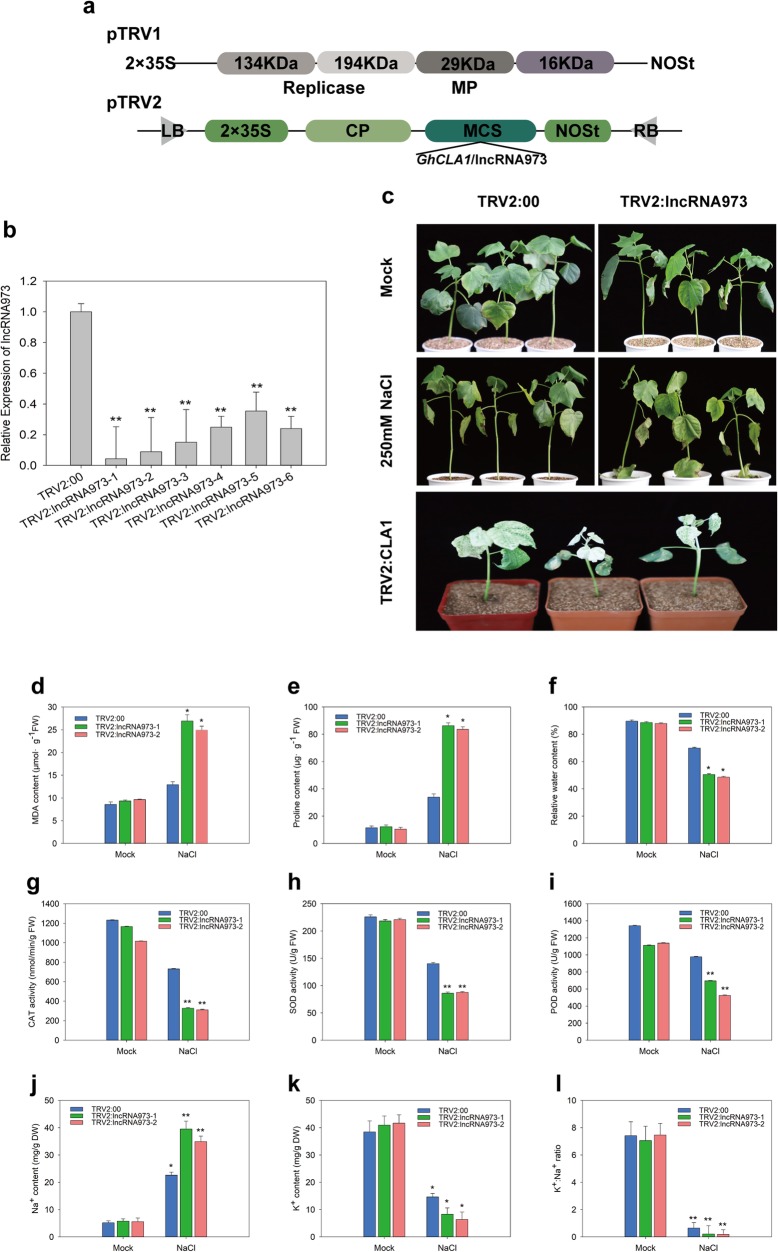
Functional identification of lncRNA973 towards salt stress using a virus-induced gene silencing (VIGS) method. **a**. Construction of the TRV2:*GhCLA1* and lncRNA973 VIGS vectors. NOS, used as resistance selection. **b**. Relative lncRNA973 transcript levels in leaves of TRV2:lncRNA973 and control plants (TRV2:00). *GhUBQ7* was used as an internal control. Each bar value represents mean ± SD of three independent experiments. **c**. Phenotypes of the silenced lncRNA973 plants at 3-d salt treatment, showing the wilting phenotype, etiolated leaves. Mock: Means to pour the same amount of water. Silencing of the endogenous *Cloroplastos alterados 1* (*CLA1*) in cotton through TRV-mediated VIGS was used as the positive control. 10-d-old cotton seedlings (*G. hirsutum* L., cv. SN91–11) with two cotyledons were infiltrated with TRV2:*CLA1*, and plants started to display an albino phenotype in their true leaves at approximately 2 weeks later. **d-f**. MDA, relative water and Pro contents in the leaves under salt stress. **g-i**. Activities of ROS-scavenging enzymes after treatment with salt: **g**: catalase (CAT), **h**: superoxide dismutase (SOD), **i**: peroxidase (POD), respectively. **j-l**. Sodium (Na^+^) and potassium (K^+^) accumulation and K^+^/Na^+^ ratio in TRV2:00 and the TRV2:lncRNA973 plants under water and NaCl condition. Mock, with water treatment. NaCl: 250 mM NaCl treatment. *, *p* < 0.05 and **, *p* < 0.01 by Student’s *t*-test compared with untreated (Mock) or salt-treated TRV2 seedlings. Data represent means ± SD

To further explore the possible roles of lncRNA973 in cotton resistance against salt stress, we silenced lncRNA973 in *G. hirsutum* ‘SN91–11’ using virus-induced gene silencing (VIGS), which is frequently used for the analysis of gene functions. The VIGS sequences of lncRNA973 and *CLOROPLASTOS ALTERADOS 1* (*CLA1*) genes were independently cloned into a TRV2 vector (Fig. 4a), producing TRV2:lncRNA973 and TRV2:*CLA1*, respectively. TRV2:*CLA1* and the empty TRV2 vector (TRV2) were used as positive and negative controls, respectively. Approximately 490 bp fragment of lncRNA973 and 465 bp of *CLA1* were used in the VIGS system. At approximately 2 weeks post-infiltration, marker gene TRV2:*CLA1*-containing plants started to display an albino phenotype in their true leaves (Fig. 4c). The expression levels of lncRNA973 in TRV2:lncRNA973-(VIGS) and TRV2-containing (empty vector) cotton plants were assessed using qRT-PCR. As shown in Fig. 4b, the drastically reduced expression of lncRNA973 in the VIGS plants demonstrated that it had been successfully knocked down. The expression of the lncRNA973-adjacent gene Gh_D04G0660 in the genome was not significantly affected (Additional file 3: Figure S3). We chose two VIGS plants and one TRV2 (empty control) plants for the salt-stress treatment. The plants were irrigated with 250 mM NaCl, and at 3 d after the salt treatment, the VIGS plants showed more severe wilting and leaf shedding phenotypes than the control plants, and the dehydrated stalks of VIGS plants were severely brown, while the stems of TRV2 plants were still green (Fig. 4c). Thus, the VIGS lines were hypersensitive to salt-stress conditions.

Additionally, to examine the tolerance of VIGS cotton to salinity stress, we examined some physiological indicators, such as MDA, Pro content, relative water content, ROS-scavenging enzyme activity and Na^+^ and K^+^ contents. There were no differences in the physiological indicators between VIGS and TRV2 plants under normal growth conditions. The MDA content, an indicator of oxidative stress, was significantly greater in VIGS plants than in TRV2 plants under salt-stress conditions (Fig. 4d). Pro and relative water content are two important stress resistance indices. The Pro accumulation was increased in both TRV2 and VIGS plants treated with salt, but the accumulation was more significant in VIGS plants (Fig. 4e). The relative water contents in VIGS plants were significantly less than in TRV2 plants under salt-stress conditions, indicating that the water loss in VIGS plants was greater, which was consistent with the phenotype (Fig. 4f).

The activities of ROS-scavenging enzymes, such as superoxide dismutase (SOD), peroxidase (POD) and catalase (CAT), play important roles in stress responses to adversity. The activities of ROS-scavenging enzymes decreased in both TRV2 and VIGS plants treated with salt; however, VIGS cotton exhibited more significant decreases compared with the TRV2 plants (Fig. 4g). Excess Na^+^ is toxic to plants, whereas K^+^ is antagonistic to Na^+^ under salt-stress conditions [33]. There were no differences in Na^+^ and K^+^ concentrations in TRV2 and VIGS plants under normal conditions. However, when exposed to salt stress, the VIGS plants accumulated more Na^+^; conversely, the concentration of Na^+^ was lower in TRV2 plants compared with in VIGS plants (Fig. 4h). Lower levels of K^+^ in VIGS plants and greater levels in TRV2 plants were determined (Fig. 4i). The imbalance between Na^+^ and K^+^ levels led to a decrease in the K^+^/Na^+^ ratio in VIGS plants (Fig. 4j). In addition, we also detected the chlorophyll, MDA, proline content and CAT activity of WT cotton seedlings at the same growth period without injected VIGS vector (Additional file 4: Fig. S4), and found that the physiological indicators were not significantly different in WT cotton seedlings and TRV2 plants, thus excluding the influence of viral vector. These results indicated that the silencing of lncRNA973 in cotton increased the MDA and Pro contents, decreased the relative water content and ROS-scavenging enzyme activities. Additionally, it promoted K^+^ exportation and the internal Na^+^ flow, resulting in an imbalance the K^+^ and Na^+^ contents, and the reduced salt tolerance of VIGS plants. Thus, lncRNA973 knockdown plants was sensitive to salt stress, and revealed that lncRNA973 could respond to salt stress by regulating the accumulation of some related physiological indicators.

### LncRNA973 regulates the expression of genes involved in the salt stress response in cotton

**Fig. 5:**
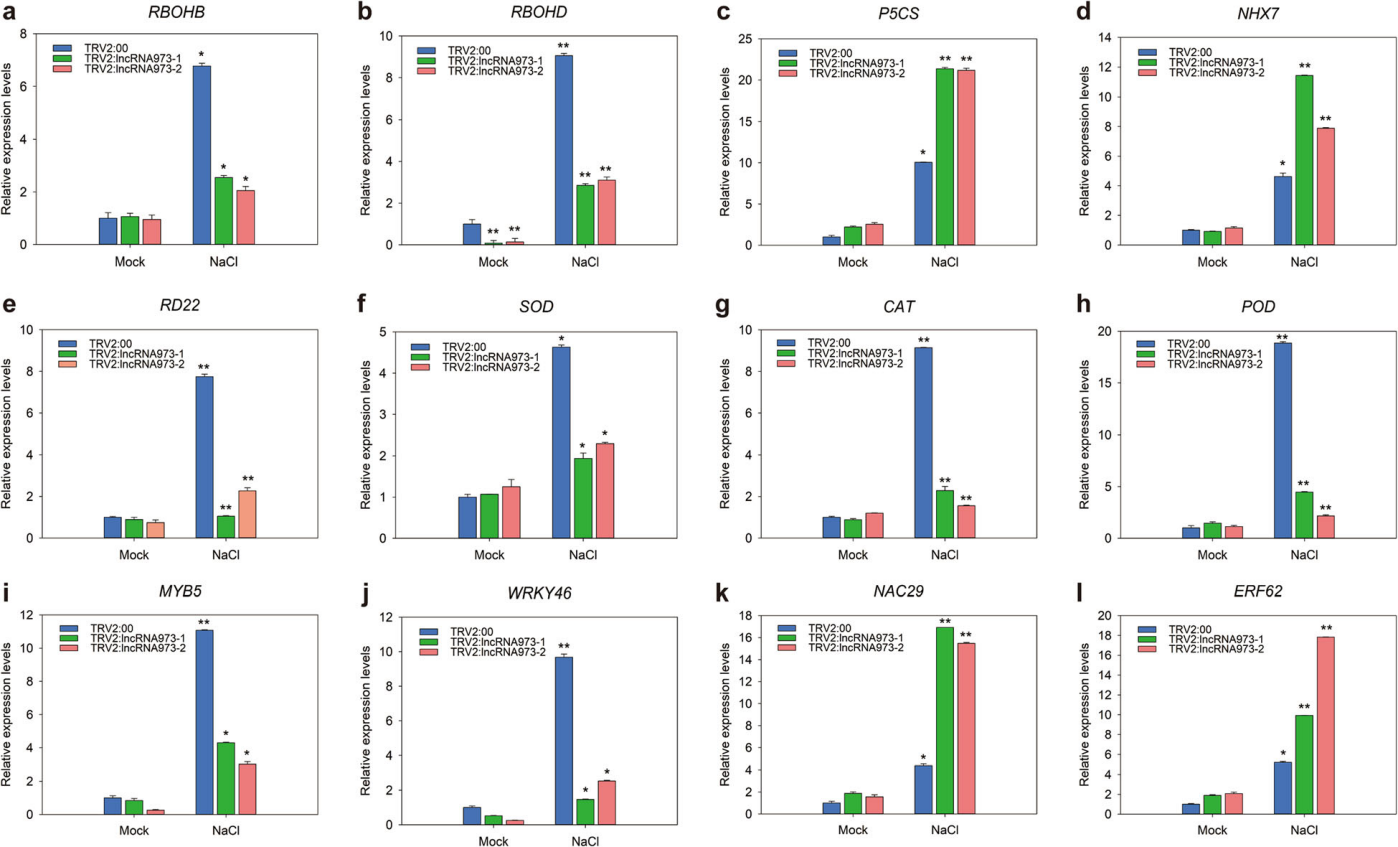
Relative expression levels of selected genes in salt-treated cotton seedlings. RNA was extracted from VIGS and TRV2 cotton seedlings leaves, and gene expression levels were measured by RT-qPCR normalized against the *GhUBQ7* gene. Relative expression levels are shown for **a.** *RBOHB*, **b.** *RBOHD*, **c.** *P5CS*, **d.** *NHX7*, **e.** *RD22*, **f.** *SOD*, **g.** *CAT*, **h.** *POD*, **i.** *MYB5*, **j.** *WRKY46*, **k.** *NAC29*, **l.** *ERF62*. Mock, without salt treatment, NaCl: 250 mM NaCl treatment. *, *p* < 0.05 and **, *p* < 0.01 by Student’s *t*-test compared with untreated (Mock) or salt-treated TRV2 seedlings. Data represent means ± SD

Based on the previous transcriptome data, the co-expression relationship and binding free energy between lncRNA973 and coding genes were calculated by RNAplex to predict co-expression genes, which included transcription factors and stress-related genes (Additional file 6: Table S1). To analyze the potential functions of lncRNA973, we selected protein-coding genes with co-expression coefficients above 0.95 with lncRNA973 for analysis. To further validate the expression of known genes critical for plant salt-stress responses, total RNA was extracted from leaves of TRV2 and VIGS salt-stress seedlings and leaves of untreated seedlings, and then, analyzed by real-time PCR. The expression levels of respiratory burst oxidases B (ROBHB) and D (ROBHD), two NADPH oxidase genes, decreased more than 30 and 50%, respectively, in salt-treated VIGS plants; however, there were no significant changes in expression levels in untreated plants (Fig. 5a, b). The induced expression of *Δ*^*1*^*-PYRROLINE-5-CARBOXYLATE SYNTHETASE* (*P5CS*) under salt-stress conditions could promote the accumulation of Pro [34]. The expression of *P5CS*, increased twofold in salt-treated VIGS plants compared with TRV2 lines (Fig. 5c). These results were consistent with the Pro physiological index results. We further assessed sodium-potassium ion-related genes in response to salt stress. Sodium hydrogen exchanger 7 (*NHX7*) encodes a vacuolar Na^+^/H^+^ antiporter involved in salt resistance that acts in the electroneutral exchange of protons for cations, such as Na^+^, across the plasma membrane [35]. *NHX7* was more highly expressed in VIGS plants than TRV2 plants under salt-stress conditions (Fig. 5d). *RESPONSIVE TO DEHYDRATION 22* (*RD22*) is activated by the abscisic acid (ABA) signaling associated with salt-stress responses [36]. Its expression also decreased in salt-treated VIGS plants (Fig. 5e). In addition, we analyzed the expression levels of ROS-related genes, *SOD*, *POD* and *CAT*. Their expression levels were significantly inhibited in VIGS plants exposed to salt stress but increased in control plants (Fig. 5f-h). Meanwhile, we examined the expression levels of homologous genes of these genes in WT and OX-lncRNA973–2/5 *Arabidopsis* plants. Under untreated conditions, the expression levels of these salt-stress related genes in OX-lncRNA973 plants were similar to those in WT *Arabidopsis* (Additional file 5: Figure S5), except that *AtPOD* gene showed significant up-regulated expression. However, the expression of these genes increased in both WT and OX-lncRNA973 plants after salt stress. The *AtRBOHB*, *AtRBOHD, AtPOD*, *AtCAT*, *AtRD22*, *AtRD29A*, and *AtRD29B* (Additional file 5: Figure S5a, b, f-j) expression levels in OX-lncRNA973 plants were significantly higher than that of WT after salt treatment, especially, *AtPOD*, *AtRD29A*, and *AtRD29B*. The expression of *AtP5CS1* and *AtNHX7* in OX-lncRNA973 plants were lower than those that of WT (Additional file 5: Figure S5c-d). These data indicated that lncRNA973 could regulate plant tolerance to salt stress by modulating the expression of genes involved in salt-stress responses.

Additionally, transcription factors may be involved in the regulation of lncRNA973. These transcription factors include, MYB5, WRKY46, NAC29 and ethylene-responsive 62 (ERF62) transcription factors. The co-expression coefficients between the transcription factors and lncRNA973 were generally greater than 0.99, indicating possible regulatory relationships between them. RT-qPCR was used to detect the expression of these transcription factors in salt-treated and untreated plants. The expression levels of MYB5 and WRKY46 were lower in VIGS plants than control plants under salt-stress conditions (Fig. 5i-j). NAC29 and ERF62 were expressed highly in salt-treated plants compared with untreated plants (Fig. 5k-l). Similarly, we also examined the expression levels of homologous genes of these transcription factors in OX-lncRNA973 plants and WT, and found that the transcription factors AtERF and AtMYB5 did not change significantly before and after treatment (Additional file 5: Figure S5k-l). The expression of AtWRKY46 increased in the normal growing OX-lncRNA973 plants, and it was obviously up-regulated in OX-lncRNA973 plants after salt treatment (Additional file 5: Figure S5 m). The expression of AtNAC3 was unchanged in the normal growing plants. Compared with WT, the expression of NAC3 in OX-lncRNA973 plants was significantly induced after salt treatment, and the expression was significantly higher than that of WT (Additional file 5: Figure S5n). Thus, lncRNA973 appears to respond to salt stress by regulating multiple genes and multiple complex mechanisms.

### LncRNA973 affects miR399 and its target PHO2 expression

**Fig. 6:**
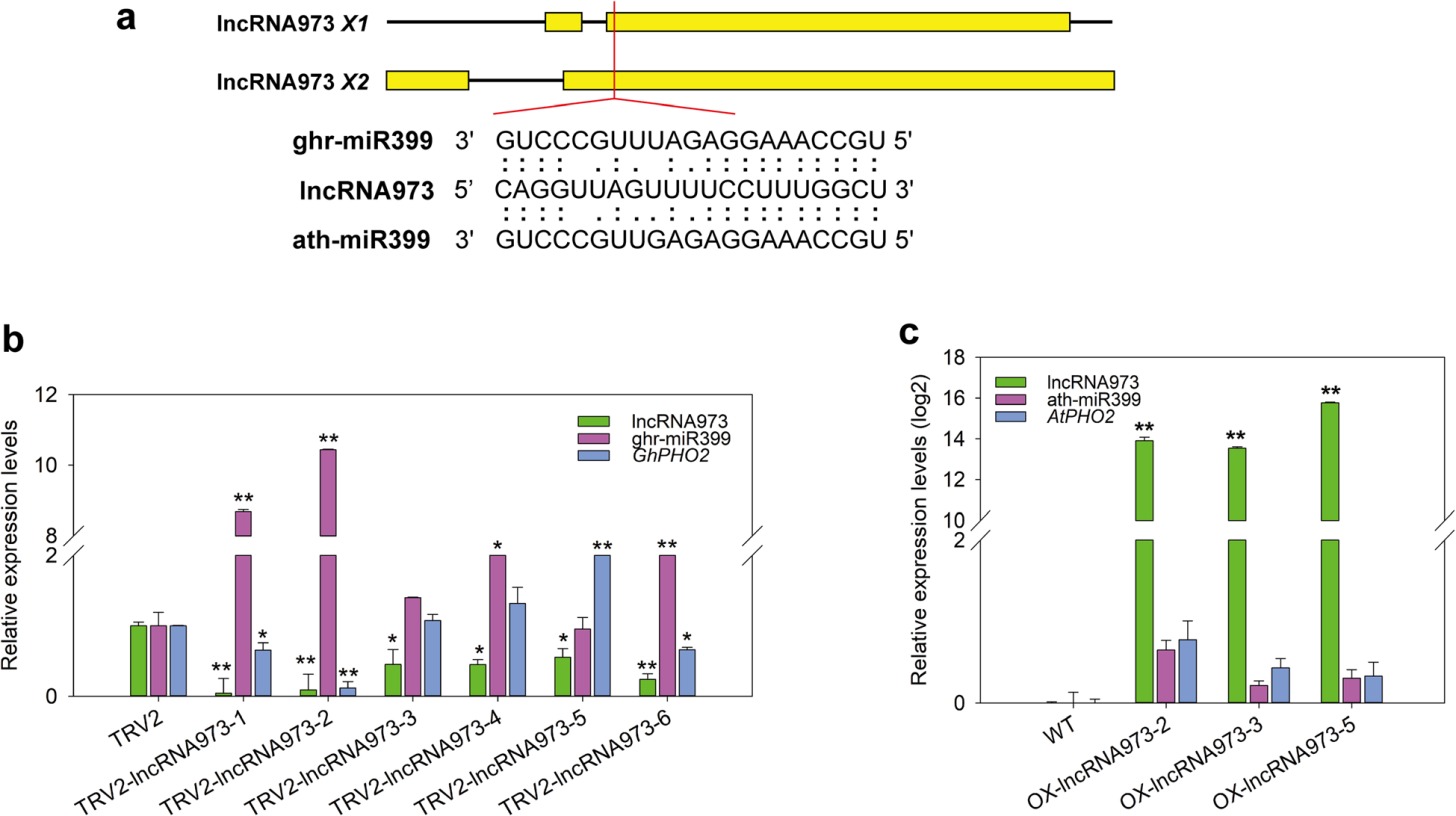
Relative expression levels of miRNA399 and lncRNA973 in VIGS and overexpression seedlings. **a**. Schematic diagram of the interaction site between ghr-miR399 and lncRNA973 transcript. **b**. The expression levels of lncRNA973, ghr-miR399, and *GhPHO2* in TRV2:00 and TRV2:lncRNA973 cotton seedling roots. **c**. The expression levels of lncRNA973, ath-miR399, and *AtPHO2* in WT and OX-lncRNA973 plants. *, *p* < 0.05 and **, *p* < 0.01 by Student’s *t*-test compared with TRV2 plants. Data represent means ± SD

Previously, we predicted that lncRNA973 may have a regulatory effect with miR399 [31]. The interaction region of lncRNA973 and miR399 is located in the second exon (Fig. 6a). Here, in order to further determine the regulatory effect of lncRNA973 on miR399, miR399 and its target gene PHO2 expression were detected in OX-lncRNA973 *Arabidopsis* and TRV2:lncRNA973 cotton plants, respectively. We detected the expression of ghr-miR399 in the knockout lncRNA973 cotton seedling roots. When the knockdown efficiency of lncRNA973 was 60% or more, the ghr-miR399 expression level significantly increased, while its target *GhPHO2* expression was significantly inhibited. And when the knockdown efficiency was less than 60%, ghr-miR399 and *GhPHO2* expression levels were not significantly affected (Fig. 6b). However, the overexpression of lncRNA973 did not significantly inhibit the expression of ath-miR399, nor did the expression of *AtPHO2* significantly change in OX-lncRNA973 *Arabidopsis* plants (Fig. 6c). Thus, lncRNA973 regulates the expression of ghr-miR399 and its target gene *GhPHO2* in cotton, while no relationship between them is found in *Arabidopsis*.

## Discussion

Recently, with the rapid development of biotechnology, many lncRNAs have been identified. However, only a few of their regulatory mechanisms have been studied, and even less is known about how these mechanisms are involved in salt-stress responses. In this study, we identified a novel lncRNA, lncRNA973 that was associated with responses to salt stress in cotton and conducted a preliminary study of its function and mechanism of action. Most intergenic lncRNAs are polyadenylated and primarily transcribed by Pol II, but some are transcribed by Pol IV and/or Pol V in plants [4, 7, 37]. The 3′ end of lncRNA973 contains a poly A tail, as in protein-encoding genes, which indicates that lncRNA973 may be transcribed from RNA Pol II. Its structure is also similar to that of protein-encoding genes, having exons and introns. An identifying characteristic of lncRNAs is that they cannot encode proteins; however, in recent years, it was determined that lncRNAs can encode polypeptides. Whether lncRNAs have coding functions can be analyzed and predicted by several different online software programs [38–40]. Therefore, we determined that lncRNA973 has no protein-encoding function, acting strictly as a lncRNA. Like many other lncRNAs, the basal transcript level of lncRNA973 was very low [31], but its expression was increased after the salt treatment. In animals and plants, only a few lncRNAs are partially evolutionarily conserved, and most lncRNAs are not homologous among species, suggesting that lncRNA is poorly conserved [41, 42]. Through conservation and evolutionary analyses, it was determined that lncRNA973 was a new gene evolving from *G. raimondii* and *G. arboreum* that is extremely poorly conserved in other species and has essentially no homologous sequences. Moreover, the characteristic subcellular localizations of lncRNAs may be connected to their functions [32, 43]. Our experiments indicated that lncRNA973 was mainly localized in the nucleus, providing the basis for our study of lncRNA973’s the function and mechanism of action.

The functional studies involved the knockdown and overexpression of lncRNA. Qin et al. studied the function of *DRIR* and found that it is a functional lncRNA that responds to salt, drought and abscisic acid. *DRIR*’s overexpression can improve tolerance to salt and drought stress [22]. The overexpression vector containing lncRNA973 was constructed and transformed into WT *Arabidopsis*. Then, T3 transgenic *Arabidopsis* was treated with NaCl solution of different concentrations. The seed germination rates, root lengths and fresh weights of lncRNA973-overexpression lines were clearly greater than of WT under salt-stress conditions. Thus, plants overexpressing lncRNA973 were more tolerant to salt stress. Additionally, we used VIGS technology to knock-down the expression of lncRNA973 in upland cotton. VIGS had been used to research the functions of lncRNAs in *Verticillium wilt* and fiber development, and the lncRNAs GhlncNAT-ANX2 and GhlncNAT-RLP7, and XLOC_545639, XLOC_039050 and XLOC_079089 respond to *Verticillium wilt* and fiber development, respectively [27, 30]. We used VIGS technology to study the function of lncRNA973 and observed the phenotypes of VIGS plants after salt treatments. LncRNA973-silenced seedlings were less tolerant to salt stress. The overexpression and knockdown experiments indicated that lncRNA973 was positively regulating cotton salt stress.

LncRNAs are key regulators of gene expression. Many lncRNAs respond to different stresses in plants. LncRNAs can directly regulate the expression of DNA, protein and miRNA, and can also indirectly regulate the expression of other genes. ELENA1 has also been found to bind to MED19a to regulate *PRI* expression in response to pathogen stress and to increase plant immune responses [21]. Here, we demonstrated that lncRNA973 regulated plant responses to salt stress by modulating the expression of a series of genes. Several key salt-related genes were investigated using RNA-seq, and we detected their expression in VIGS silenced lncRNA973 seedlings. The expression levels of some salt-related genes positively regulated were dramatically inhibited, while other negatively regulated genes were significantly induced. The transcription of the ROS-scavenging genes *SOD*, *CAT* and *POD* were downregulated in lncRNA973-silenced plants, and SOD, POD and CAT activities in salt-treated VIGS plants were significantly inhibited. This inhibition was positively correlated with the expression level. Ibrahim et al. found that SOD, POD and CAT activities were inhibited in the salt-sensitive ‘Zhongmian’ 41 compared with salt-tolerant ‘Zhongmian’ 23 during salt treatments [44]. These results were consistent with our findings that the activities of the three enzymes in salt-sensitive materials were reduced. Plant ROBH, a homolog of NADPH oxidases, has a crucial role in plant’ stress responses. RBOH regulates ROS-dependent ion regulation and plays a role in homeostasis in response to salt stress [45]. In our experiments, we found that the expression levels of *RBOHB* and *RBOHD*, two members of the RBOH family, were reduced in salt-stressed VIGS plants, which reduced the plant’ salt tolerance. Ion homeostasis in plants is also critical for salt tolerance. An excessive Na^+^ accumulation reduces the tolerance of plants and decreases growth and development. In salt-stressed VIGS plants, excess Na^+^ was accumulated and the K^+^/Na^+^ ratio decreased, while in the control plants, the K^+^/Na^+^ ratio increased. The expression of *NHX7* was affected in lncRNA973-knockout cotton and was regulated by lncRNA973. These were consistent with the findings of Gao et al. [46]. *P5CS* can be considered a marker gene that responds to salt stress [47]. The higher level of *P5CS* suggested that silencing lncRNA973 in cotton decreased degree of salt stress, whereas the expression of *AtP5CS1* was inhibited in OX-lncRNA973 plants. Transcription factors, especially MYB, NAC, ERF and WRKY, play important roles in salt-stress responses. MYB and WRKY mainly positively or negatively regulate gene expression in response to salt stress, and the NAC and ERF families participate in senescence in response to drought and salt stresses. In our experiment, after silencing lncRNA973, the expression levels of MYB5 and WRKY46 decreased, while those of NAC29 and ERF62 significantly increased, suggesting that they may be located downstream of lncRNA973 as well as *SOD*, *CAT*, *POD* and *P5CS*, which are regulated by lncRNA973.

MiRNA has an important role in lncRNA. Studies in plants have indicated that lncRNAs are endogenous mimetic targets of miRNAs, which directly inhibit miRNA regulation of mRNA by binding to miRNAs. This decreases the expression of miRNAs, resulting in an increase in the mRNA content [48–50]. Jalali et al. reported that lncRNAs not only have potential miRNA regulatory elements, but also participate in the miRNA regulatory network [51]. We found that lncRNA973 may have a regulatory effect with miR399. The expression of ghr-miR399 was detected in TRV2:lncRNA973 cotton, and it was found that the expression of ghr-miR399 was increased after knocking out lncRNA973, and its target gene *GhPHO2* expression was relatively inhibited. However, we did not find such results in OX-lncRNA973 *Arabidopsis*. Thus, there may be a regulatory mechanism or dose effect of endogenous competitive RNA between the two, but the specific regulatory relationship is still unknown.

**Fig. 7:**
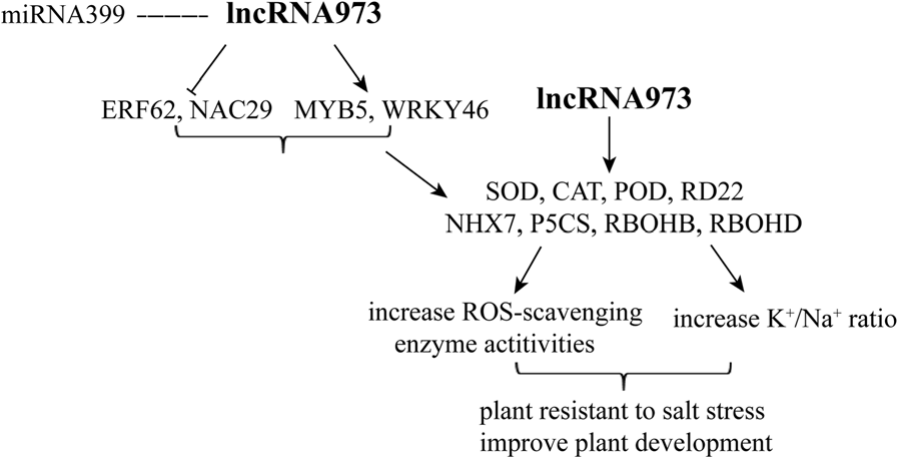
Possible regulation model of the lncRNA973 responses to salt-stress pathway. The lncRNA973 acts as a positive regulator that responses to salt-stress in cotton. LncRNA973 may increase the salt tolerance of cotton by inducing the expression of transcription factors *MYB5*, *WRKY46*, *ERF62*, *NAC29* and other downstream genes *SOD*, *CAT*, *POD*, *NHX7*, *P5CS*, *RBOHB*, *RBOHD*, which are important salt-responsive genes. Arrows indicate facilitating expression and dotted line indicates an undetermined regulatory relationship

Our results indicated that lncRNA973’s overexpression can enhance the salt tolerance of plants, while lncRNA973’s knocked-down expression can reduce salt tolerance and affect the expression of some genes involved in responding to salt stress. In addition, lncRNA973 may also participate in the regulation of miRNAs. These results allowed us to propose a model of the mechanism by which lncRNA973 regulates gene responses to salt stress (Fig. 7). The transcription factors MYB5, WRKY46, NAC29 and ERF62 may be regulated by lncRNA973. In addition, some ROS-scavenging genes and genes that regulate Na^+^ and K^+^ accumulation were inhibited, and the expression of Pro-synthesizing genes was increased. These genes could be regulated indirectly by lncRNA973. *SOD*, *CAT*, and other genes were regulated by these transcription factors. LncRNA973 may increase the salt tolerance of cotton by inducing the expression of ROS-scavenging genes and Na^+^ and K^+^ accumulation-related gene.

## Conclusions

In this study, we identified and characterized the functions of a salt stress-related lncRNA, lncRNA973. The structure of lncRNA973 is similar to mRNA and its conservation is poor. In situ hybridization analysis revealed that lncRNA973 localized mainly to the nucleus, the expression level was significantly increased by salt treatments. Here lncRNA973 was transformed into *Arabidopsis* and overexpressed, the transgenic plants enhanced tolerance to salt stress, while decreasing its expression had reduced salt tolerance. The silencing of lncRNA973 in cotton increased the MDA and Pro contents, decreased the relative water content and ROS-scavenging enzyme activities, and resulted in an imbalance the K^+^ and Na^+^ contents, and the reduced salt tolerance of VIGS plants. LncRNA973 could respond to salt stress by regulating the accumulation of some related physiological indicators. A transcriptome data analysis indicated that lncRNA973 may be involved in the regulation of genes primarily enriched in stress-related biological processes and pathways [52]. These included reactive oxygen species-scavenging genes, transcription factors and genes involved in salt stress-related processes. Real-time quantitative PCR confirmed that knockdown lncRNA973 affects the expression of these genes. LncRNA973 responses to salt stress by modulating the expression of some salt stress-related genes. Moreover, these results have deepened the understanding of the salt tolerance mechanism of cotton, and provided new ideas for the identification of salt tolerance and the breeding of new salt-tolerant varieties.

## Methods

### Plant materials, growth conditions and stress treatment

In this study, the cultivar *G. hirsutum* SN91–11, a salt-tolerant cultivar, which was selected and bred by our laboratory and is currently kept in the laboratory. Its physiological properties are consistent with the previous description [31]. Upland cotton (*G. hirsutum* ‘SN91–11’) seeds were germinated in a mixture of peat and vermiculite at 28 °C. Cotton seeds were grown under a 16-h/8-h light/dark cycle at 28 °C/22 °C. Transgenic and WT *Arabidopsis* (Col-0 ecotype, preserved by our laboratory) seeds were surface-sterilized using 70% alcohol for 5 min and 2% NaClO for 5 min, then washed five times with sterile ddH_2_O and germinated on 1/2 Murashige-Skoog (MS) medium. Seeds were first vernalized for 3d at 4 °C in the dark and then germinated in a growth chamber at 22 °C, 16-h /8-h light/dark photoperiod.

Upland cotton ‘SN91–11’ plants were grown under standard field conditions. For the tissue/organ-specific expression profiling analysis, cotyledons, stems and roots were harvested from 10-d-old seedlings. Cotton seedlings were subjected to different abiotic stress treatments after the expansion of the first true leaf. Uniformly cultured cotton seedlings were cultivated in Hoagland’s solution supplemented with 250 mM NaCl. The roots, stems, and leaves of the cotton seedling were collected at designated time points (0, 1, 3, 6, 12 and 24 h). All the tissues/organs were frozen in liquid nitrogen and stored at − 80 °C until total RNA was extracted.

### LncRNA973 targets prediction and annotation

The potential target genes of lncRNAs are predicted according to their regulatory methods, which are divided into *cis*- and *trans*-acting. *Trans* regulation is not dependent on positional relationships, and RNAplex [53] is used to screen *trans*-target genes by calculating the binding free energy of lncRNA973 and its co-expressed protein-coding genes (Additional file 6: Table S1). Predictive targets are screened according to free energy and correlation, and the screening criterion Cor > 0.90.

### Quantitative real-time PCR analysis

For the qRT-PCR analysis, total RNA was isolated from plants using RNAprep Pure Plant Kit Polysaccharides & Polyphenolics-rich (TIANGEN Biotech, Beijing, China). The RNA concentrations were quantified using a NanoDrop ND-2000 spectrophotometer (Thermo Fisher Scientific, Wilmington, DE, USA). The RNA was used for first-strand cDNA synthesis with a the PrimeScript RT-qPCR kit (TaKaRa, Dalian, China), reverse transcription primers are a mixture of random primers and oligo dT, and the cDNA was then used as a template for lncRNA and mRNAs qRT-PCR amplification. qRT-PCR reactions were performed using SYBR *Premix Ex Taq* (Tli RNaseH Plus) (TaKaRa) and the Applied Biosystems™ QuantStudio™ 6 Flex Real-Time PCR System (ABI; Life Technologies Inc., Burlington, ON, Canada). The reaction was performed using a two-step method. The step was 95 °C for 30 s as the initial denaturation, the second step was 40 cycles of 95 °C for 5 s and 60 °C for 30 s for amplification in a final volume of 20 μL, which contained 2 μL of a 1/10 dilution of cDNA in water, 10 μL of the 2 × SYBR *Premix Ex Taq*, 0.5 μM concentrations of forward and reverse primers, and ddH_2_O. The kits used for detecting miRNA expression were Mir-X™ miRNA First-Strand Synthesis and TB Green™ *Premix Ex* Taq™ II (Tli RNaseH Plus) (TaKaRa). The miRNA specific primers were designed based on mature miRNA sequences as 5′ primers for qRT-PCR (Additional file 7: Table S2), and the 3′ primers for qRT-PCR were mRQ 3′ primers provided in the Mir-X™ miRNA First-Strand Synthesis kit. The experimental steps were carried out according to the kit instructions. All reactions were run in triplicate, and controls (no template and no RT) were included for each gene. Cotton *UBQ7* and *Arabidopsis Actin2* were used as normalizers and the relative expression levels of genes were determined using the 2^-∆∆ct^ method [54, 55]. The primer pairs used for qRT-PCR are listed in Additional file 7: Table S2. All the above samples were performed three replicates, and the data were analyzed using Student’s *t-*test.

### RNA in situ hybridization

RNA in situ hybridization probes were designed as previously described by Duncan et al. [56], and each sequence was detected in the cotton genome to determine the specificity of the target probe (Additional file 7: Table S2). Roots were taken from 10-day-old cotton seedlings and placed into glass dishes containing 10 mL fixation buffer prepared with diethyl pyrocarbonate (DEPC) (1:1:18 formalin-glacial acetic acid-70% ethanol) to fix for 2–12 h at room temperature.

After fixation, the tissue was dehydrated with gradient alcohol and then soaked in wax, embedded and sectioned. Paraffin sections were baked at 62 °C for 2 h, and then placed in xylene and alcohol of different concentrations for dewaxing. Tissue samples were digested with proteinase K (Servicebio, WuHan, China) at 37 °C, and washed with phosphate buffer saline (PBS) (Servicebio) three times for 5 min each. The pre-hybridization solution was added and incubated at 37 °C for 1 h, Then, 100 μL of hybridization solution, containing the probes at final concentrations of 250 nM, was added to the pre-hybridization solution of each slide, and the slides were hybridized at 37 °C overnight in the dark. The hybridization solution containing the unhybridized probe was removed using a pipette in the morning. After hybridization, the slides were first washed with 2 × SSC at 37 °C for 10 min, then washed with 1 × SSC at 37 °C for two times for 5 min each, and finally washed with 0.5 × SSC at room temperature for 10 min. Then bovine serum albumin (BSA) was added and the slides were incubated for 30 min at room temperature. Finally, after the hybridization solution was removed, anti-DIG-488 was added to the slides and incubated at 37 °C for 50 min before washing with PBS four times for 5 min each. 4′,6-diamino-phenylindole (DAPI) dye solution was added to the slides, and then, they were incubated for 8 min in the dark. Anti-fluorescence quenching sealing tablets were added and sealed after washing.

An automatic slice scanning system, 3D HISTECH, was used for imaging. The following wavelengths were used for fluorescence detection: for DAPI, an excitation of 330–380 nm and signal detection of 420 nm; for probes labelled with DIG an excitation of 465–495 nm and signal was detection of 515–555 nm. Maximum projections and an analysis of three-dimensional pictures were performed using CaseViewer (an implementation of 3DHISTECH, a public domain program available at https://www.3dhistech.com/caseviewer).

### Isolation and extraction of nuclear-cytoplasmic RNA

The nuclear and cytoplasmic samples were isolated from the root tips of 10-day-old cotton seedlings. Firstly, protoplasts of root tips were prepared, and then protoplasts were used to separate the nucleus and cytoplasm. The preparation of protoplasts and the separation of nuclear and cytoplasmic were mainly based on Nuclear and cytoplasmic isolation kit instructions (Bestbio Biotech Inc., Shanghai, China). RNAprep Pure Plant Kit Polysaccharides & Polyphenolics-rich (TIANGEN) kit was used to extract the respective RNA from nucleus and cytoplasm, respectively. Reverse transcription and RT-PCR were performed by referring to the above qRT-PCR process. *U6* and tRNA were used as internal controls for nuclear and cytoplasmic extracts, respectively.

### lncRNA973 cloning, vector construction, genetic transformation

Using the previously obtained transcribed sequence, two specific primers (Additional file 7: Table S2) based on the consensus sequence of cotton leaves were designed for lncRNA973 cloning and amplification. The cDNA library was obtained by PrimeScript™ II 1st Strand cDNA Synthesis Kit (TaKaRa) with reverse transcription primers oligo dT. Full-length lncRNA973 was obtained by PCR using PrimeSTAR® HS DNA Polymerase (TaKaRa) with this pair primer. The PCR product was separated by agarose gel electrophoresis on a 1% agarose gel, which was then stained with ethidium bromide, purified using the TIANgel Midi Purification Kit (TIANGEN Biotech, Beijing, China), ligated into the pMD19-T vector (TaKaRa), and confirmed by DNA sequencing. The lncRNA973 transcript sequence was inserted downstream of the *Ubi* promoter in the recombinant binary vector pCAMBIA1300-GFP using the *Kpn* I and *Pst* I restriction loci. The constructs were transferred into *A. tumefaciens* strain GV3101 and transformed into WT *Arabidopsis* using the floral dip method. Transgenic lines were selected on 1/2 MS medium supplemented with 50 mg/L hygromycin. Independent transgenic lines were obtained, plant DNA was extracted, and PCR was performed to amplify and authenticate the lncRNA973 sequences transformed into these transgenic *Arabidopsis* plants. Three T3 homozygous lines were selected and used for subsequent salt-stress tolerance tests.

### Stress-treated transgenic *Arabidopsis*

For NaCl treatment, seeds were stratified for 2d at 4 °C in the dark and plated onto Petri dishes containing basal 1/2 MS medium, which was divided into control and salt treatments. The concentrations of the salt treatment were 100 and 150 mmol/L. Sterilized seeds were placed in Petri dishes on 1/2 MS medium and grown for 7 days in a culture chamber at 22 °C with 16-h/8-h light/dark photoperiod. Approximately 30–40 seedlings were used per replicate, and three replicates were used per treatment. The germination rates of the seeds were determined every day for 7 days. After 7 days of growth, the seedlings were transferred to the corresponding control and salt treatment media, and then images of each sample were taken. Primary root length, plant fresh weight and cotyledon greening were scored. The measurement of the primary root length was performed using Image J software. Fresh weights were measured using a precision balance.

### Virus-induced gene silencing (VIGS) constructs and salt treatment

The plasmid TRV2 (pYL156) and TRV1 (pYL192), which were purchased from Changsha Yingrun Biotechnology Co., Ltd., were used for VIGS. The VIGS sequence was designed using the SGN VIGS Tool (http://vigs.solgenomics.net/) and cloned into the TRV2 vector using the T4 DNA ligation cloning method. The lengths of the silencing fragments for lncRNA973 and *GhCLA1* were 490 and 465 bp, respectively. The TRV-VIGS constructs were also transformed into *A. tumefaciens* strain GV3101. Then, strain GV3101, containing the different constructs, was infiltrated into 10-day-old cotton seedling’ cotyledons according to the method described by Gao et al. [57]. TRV2:*CLA1* (*CLOROPLASTOS ALTERADOS 1*) was utilized as the positive control and the empty vector TRV2:00 was the negative control. The silencing of endogenous gene expression was detected using an RT-PCR assay. All the primers used in the VIGS experiments are listed in Additional file 7: Table S2.

Two weeks after infection, the albino leaf phenotype appeared in plants transformed with the positive control gene *CLA1*. Then, cotton plants harboring TRV2:lncRNA973 and the negative control TRV2:00 were subjected to salt treatments. When the control TRV2:00 and the transgenic TRV2:lncRNA973 plants grew ~ 6–7 leaves, similar sized plants were selected and irrigated continuously with the same volume of water or salt solution (250 mM/L NaCl). The plants were observed and photographed during the treatment.

### Analysis of chlorophyll, relative water, Pro and MDA contents

Leaves of stress-treated and normal control plants of similar developmental stages were used to measure the chlorophyll, relative water, Pro and MDA contents. The chlorophyll content of cotton and *Arabidopsis* leaves in the field was detected using ethanol and acetone extraction. Approximately 0.1 g of the leaves (with the main stem removed) were placed in a glass test tube, and 10–20 mL of a mixture of ethanol and acetone was added and shaken at room temperature for 16 h in a shaker. The chlorophyll was measured for absorbance at 645 and 663 nm in a spectrophotometer. The total chlorophyll content is calculated by the following equation: ((8.05*A663 + 20.29*A645)) *V (extraction volume)/ (weight of sample) *1000.

The relative water content was determined as described by Wang et al. [58]. Leaves of salt-treated and control plants at similar developmental stages were used for Pro content determinations. Pro was detected as described previously [59]. A Pro standard curve was prepared using 0-12 μg Pro standard solution that had been heated for 30 min at 100 °C. After cooling, 4 mL toluene was added, and the absorbance of the organic phase at 520 nm was determined by ultraviolet spectrophotometer. The Pro content of each sample was determined by comparison with the standard curve.

The MDA level was determined using the thiobarbital acid method as described previously [60]. All the above samples were performed three replicates, and the data were analyzed using Student’s *t-*test.

### Determination of POD, CAT and SOD activity

Salt-treated and untreated fresh cotton leaves (~ 0.1 g) at similar stages of development were used to detect ROS-related enzyme activities. The POD, CAT and SOD activity levels were measured and performed according to kit instructions (SuZhou Comin Biotechnology Co. Ltd., JiangSu, China).

## Supplementary information

**Additional file 1: Figure S1.** Analysis of the lncRNA973 *X1* open reading frame and molecular localization of lncRNA973. **a**. All frames (gray boxes) were identified in the three forward frames. The two longest open reading frames encoded 107 and 53 amino acids (aa). **b**. RT-PCR analysis the fragment of lncRNA973 from total RNA, nucleus RNA and cytoplasm RNA. Nuclear *U6* and cytoplasmic tRNA were used as controls. A house-keeping mRNA (*UBQ7*) was used as a negative control. (TIF 1175 kb)

**Additional file 2: Figure S2.** Expression of lncRNA973 in transgenic *Arabidopsis* and wild type (WT). **a**. T-DNA region of the plasmid pCAMBIA1300-GFP for transformation. lncRNA973 was controlled by Ubiquitin protein promoter *Ubi*. NOS, used as resistance selection. RB, T-DNA right border; LB, T-DNA left border. **b**. The full length of lncRNA973 transcript was obtained from cotton by PCR amplification. **c**. PCR analysis the transcript of lncRNA973 was inserted into the genome of *Arabidopsis*. **d**. Relative transcript levels of lncRNA973 in the WT and transgenic lines, OX-lncRNA973–2, OX-lncRNA973–3 and OX-lncRNA973–5. **, *p* < 0.01 by Student’s *t*-test. Data represent means ±SD. (TIF 740 kb)

**Additional file 3: Figure S3.** Relative expression level of the lncRNA973 neighboring gene Gh_D04G0660 in the control (TRV2:00) and TRV2:lncRNA973 mutant. (TIF 269 kb)

**Additional file 4: Figure S4.** Total chlorophyll, MDA, and Pro contents and activity of CAT in the WT, TRV2:00, and TRV2:lncRNA973 leaves under salt stress and mock. WT: Uninfected cotton. Mock, with water treatment. NaCl: 250 mM NaCl treatment. *, *p* < 0.05 and **, *p* < 0.01 by Student’s *t*-test compared with untreated (Mock). Data represent means ± SD. (TIF 491 kb)

**Additional file 5: Figure S5.** Relative expression levels of selected genes in salt-treated WT and OX-lncRNA973 *Arabidopsis* plants. RNA was extracted from WT and OX-lncRNA973 plants, and gene expression levels were measured by RT-qPCR normalized against the *Atactin* gene. Relative expression levels are shown for **a**. *AtRBOHB*, **b**. *AtRBOHD*, **c**. *AtP5CS1*, **d**. *AtNHX7*, **e**. *AtSOD*, **f**. *AtCAT*, **g**. *AtPOD*, **h**. *AtRD22*, **i**. *AtRD29A*, **j**. *AtRD29B*, **k**. *AtNAC3*, **l**. *AtWRKY46*, **m**. *AtERF*, **n**. *AtMYB5*. Mock, without salt treatment, NaCl: 200 mM NaCl treatment. *, *p* < 0.05 and **, *p* < 0.01 by Student’s *t*-test compared with untreated (Mock). Data represent means ± SD. (TIF 1397 kb)

**Additional file 6: Table S1.** lncRNA973 co-expressed protein-coding genes information. (XLS 3055 kb)

**Additional file 7: Table S2.** Gene-specific primers and probes used in the research. (XLSX 13 kb)

## Data Availability

Sequence data from RNA-seq described in this article had been released at NCBI, PRJNA416197. The genome data supporting the conclusions of this article was from CottonFGD (https://cottonfgd.org/). The results data generated during this study were included in this article and its additional files.

## Abbreviations

BSA: bovine serum albumin
*CLA1*: *CLOROPLASTOS ALTERADOS 1*
DAPI: 4′,6-diamino-phenylindole
DEPC: diethylpyrocarbonate
ELENA1: ELF18-INDUCED LONG-NONCODING RNA1
lncRNA: long non-coding RNA
MDA: malondialdehyde
MS: Murashige-Skoog
ORF: open reading frames
PBS: phosphate buffer saline
qRT-PCR: quantitative real-time PCR
ROS: reactive oxygen species
SSC: saline sodium citrate buffer
VIGS: virus-induced gene silencing
WT: wild type
