# Supplementary figures and images for: The long non-coding RNA lncRNA973 is involved in cotton response to salt stress
Source: BMC Plant Biol. 2019 Oct 30;19:459. doi: 10.1186/s12870-019-2088-0 (PMC6822370; doi:10.1186/s12870-019-2088-0)

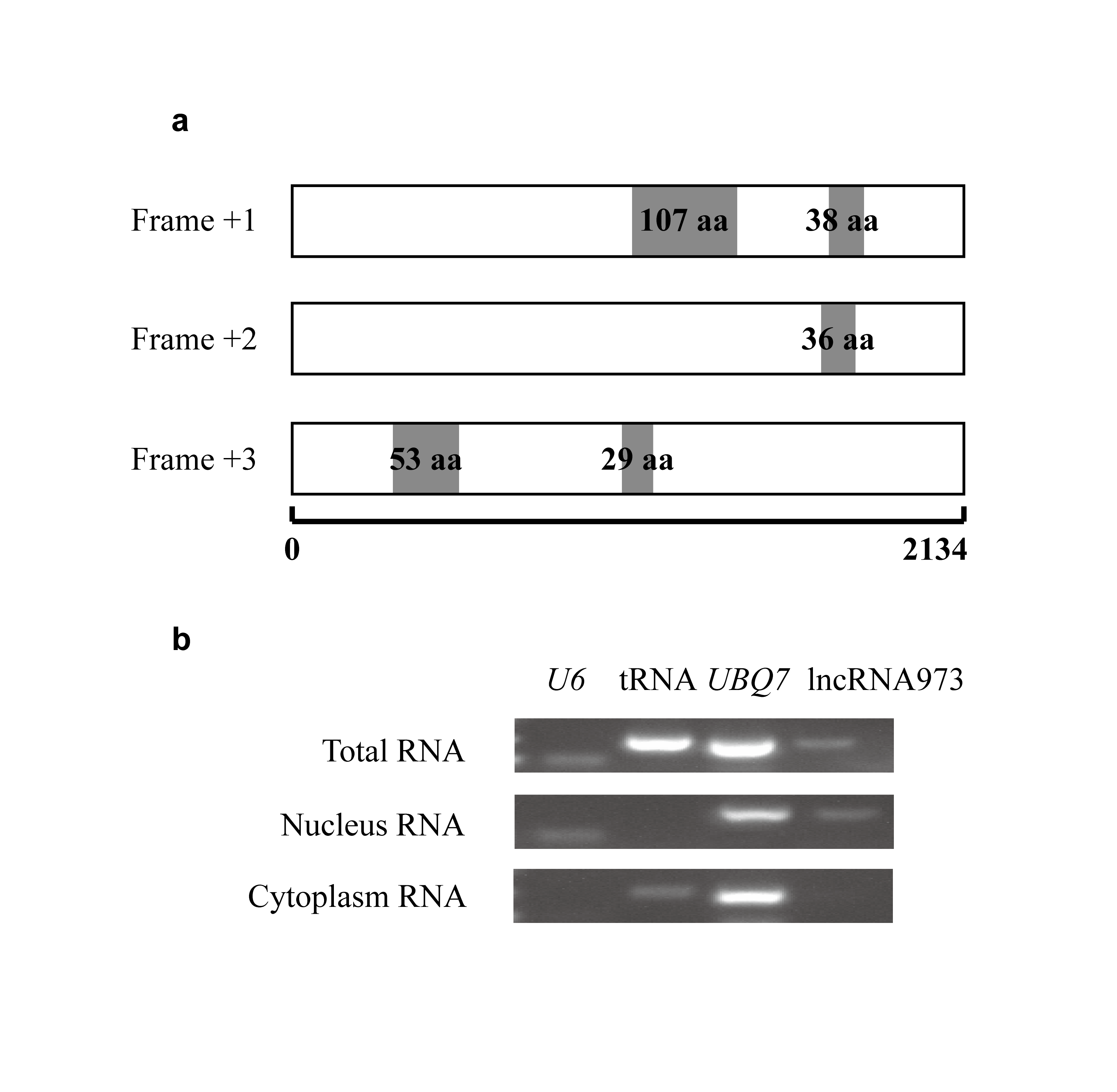

Supplement: Supplementary file 1 — Additional file 1: Figure S1. Analysis of the lncRNA973 X1 open reading frame and molecular localization of lncRNA973. a. All frames (gray boxes) were identified in the three forward frames. The two longest open reading frames encoded 107 and 53 amino acids (aa). b. RT-PCR analysis the fragment of lncRNA973 from total RNA, nucleus RNA and cytoplasm RNA. Nuclear U6 and cytoplasmic tRNA were used as controls. A house-keeping mRNA (UBQ7) was used as a negative control. (TIF 1175 kb) [file 12870_2019_2088_MOESM1_ESM.tif]

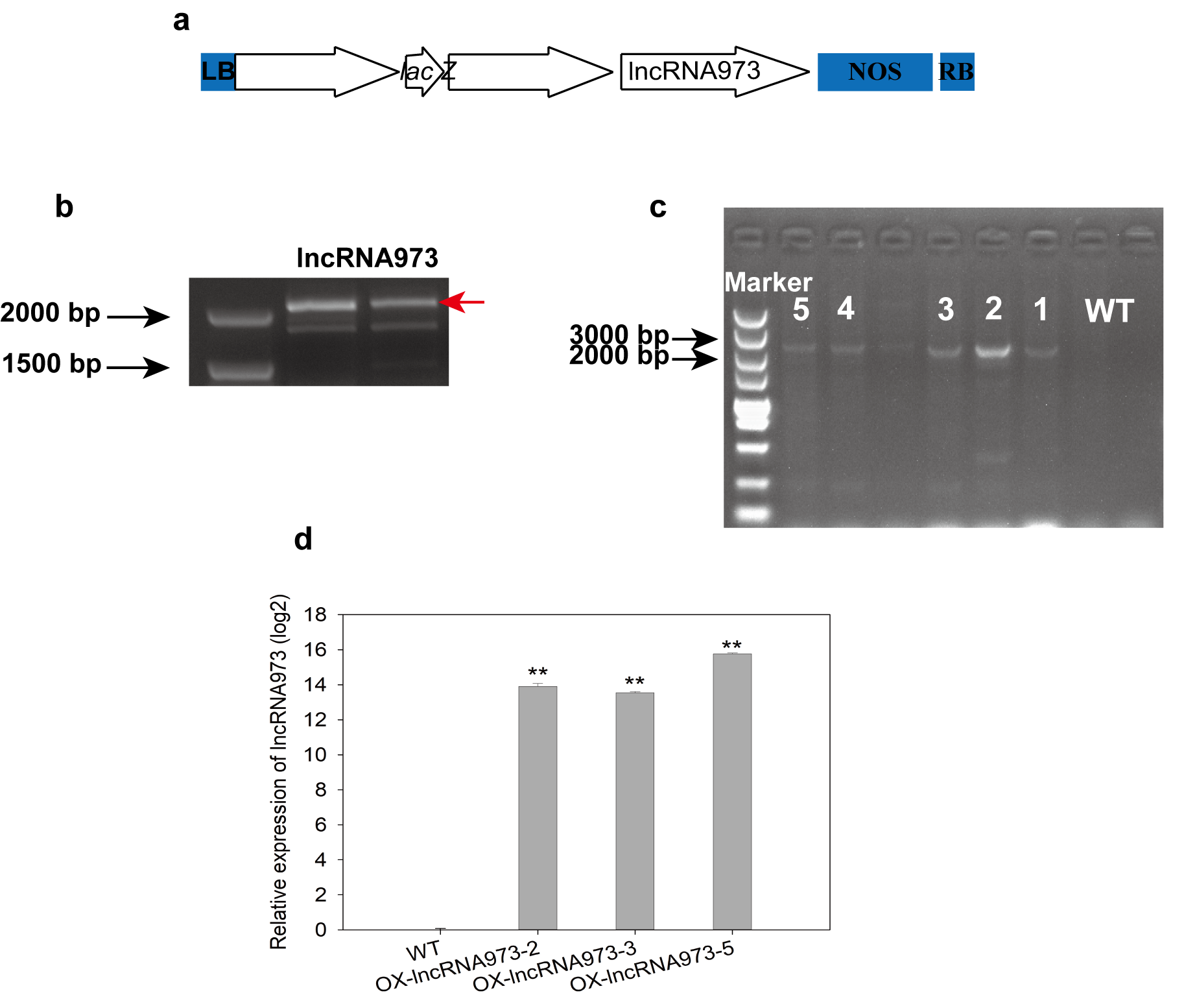

Supplement: Supplementary file 2 — Additional file 2: Figure S2. Expression of lncRNA973 in transgenic Arabidopsis and wild type (WT). a. T-DNA region of the plasmid pCAMBIA1300-GFP for transformation. lncRNA973 was controlled by Ubiquitin protein promoter Ubi. NOS, used as resistance selection. RB, T-DNA right border; LB, T-DNA left border. b. The full length of lncRNA973 transcript was obtained from cotton by PCR amplification. c. PCR analysis the transcript of lncRNA973 was inserted into the genome of Arabidopsis. d. Relative transcript levels of lncRNA973 in the WT and transgenic lines, OX-lncRNA973–2, OX-lncRNA973–3 and OX-lncRNA973–5. **, p < 0.01 by Student’s t-test. Data represent means ±SD. (TIF 740 kb) [file 12870_2019_2088_MOESM2_ESM.tif]

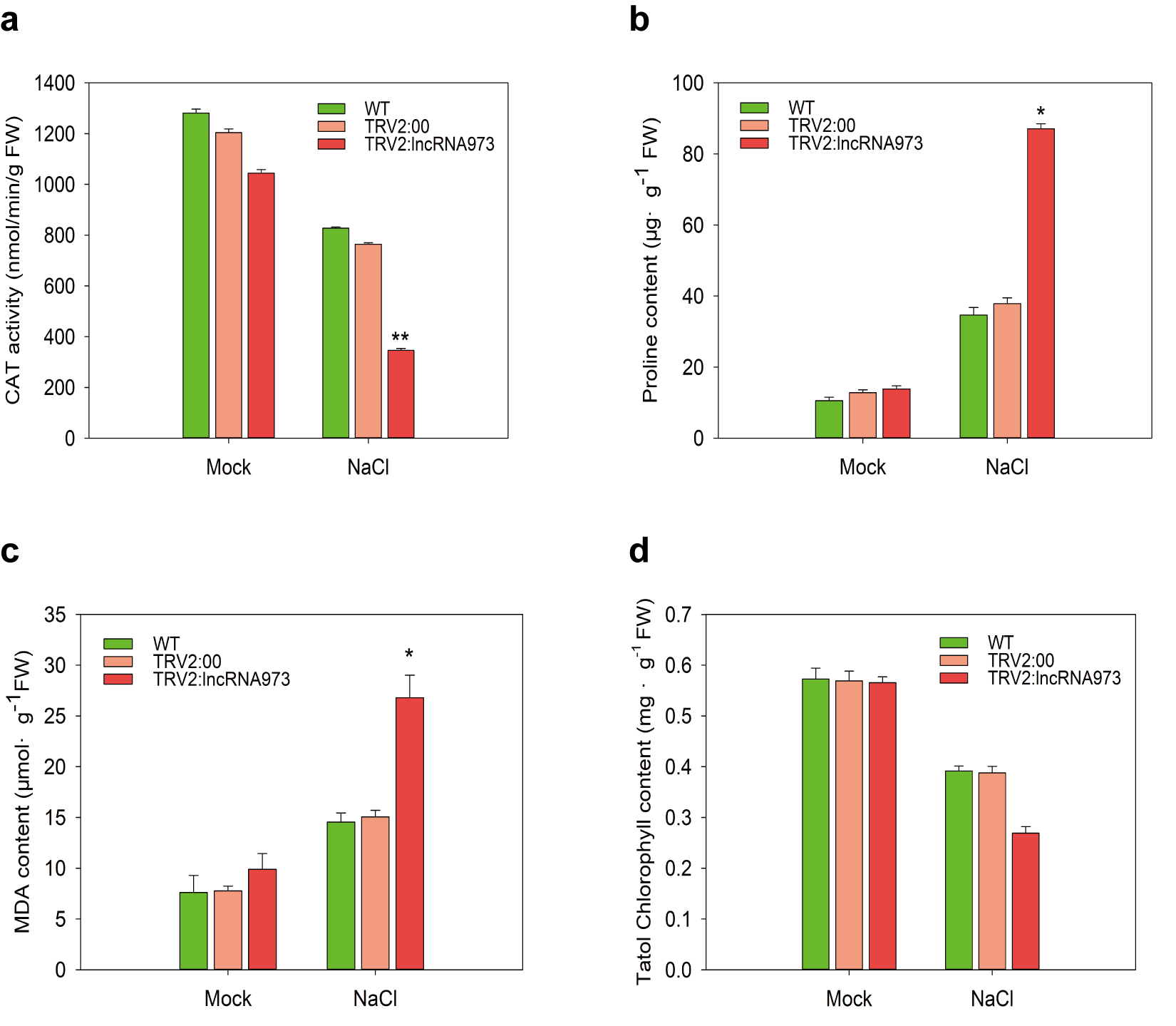

Supplement: Supplementary file 4 — Additional file 4: Figure S4. Total chlorophyll, MDA, and Pro contents and activity of CAT in the WT, TRV2:00, and TRV2:lncRNA973 leaves under salt stress and mock. WT: Uninfected cotton. Mock, with water treatment. NaCl: 250 mM NaCl treatment. *, p < 0.05 and **, p < 0.01 by Student’s t-test compared with untreated (Mock). Data represent means ± SD. (TIF 491 kb) [file 12870_2019_2088_MOESM4_ESM.tif]

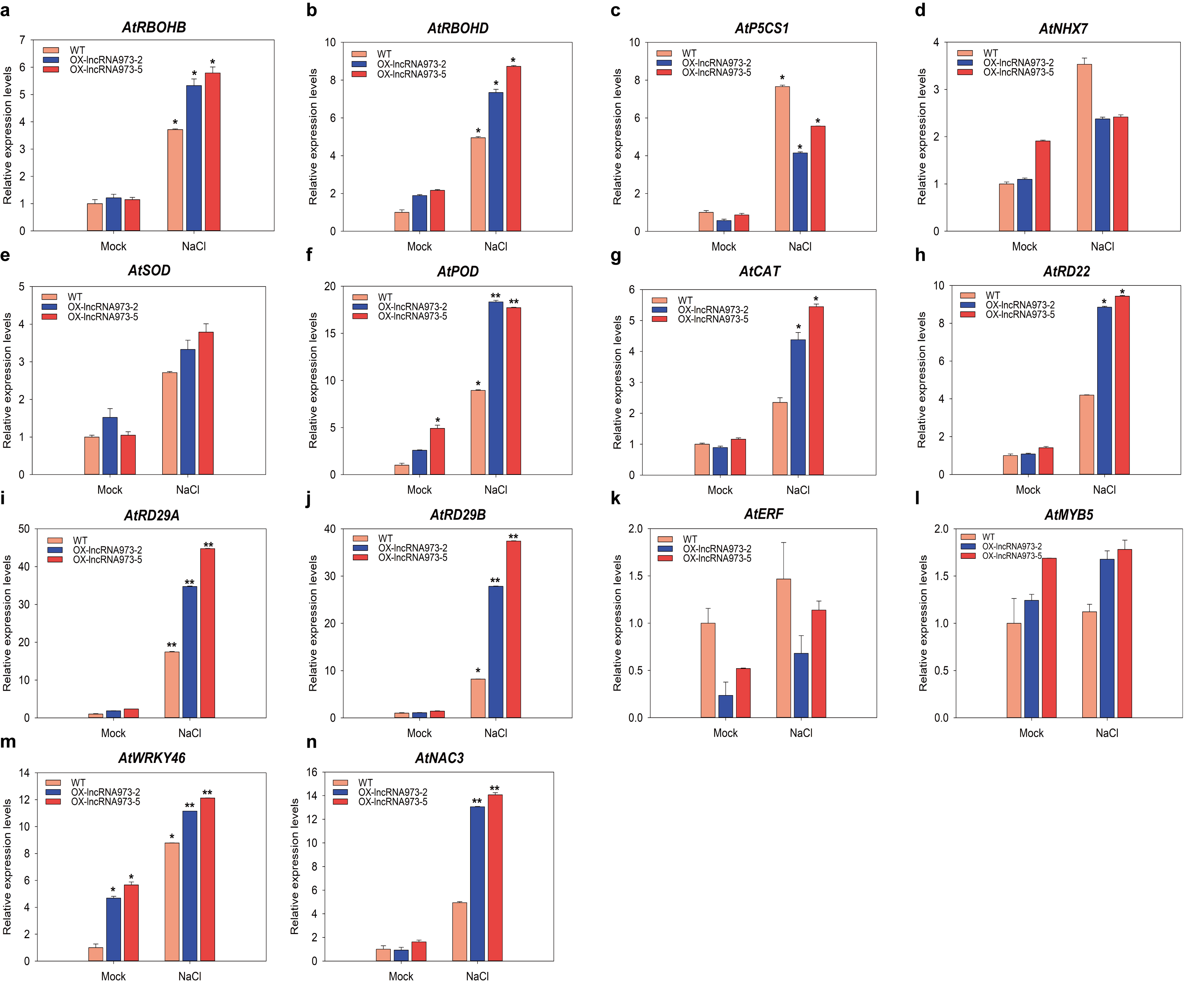

Supplement: Supplementary file 5 — Additional file 5: Figure S5. Relative expression levels of selected genes in salt-treated WT and OX-lncRNA973 Arabidopsis plants. RNA was extracted from WT and OX-lncRNA973 plants, and gene expression levels were measured by RT-qPCR normalized against the Atactin gene. Relative expression levels are shown for a. AtRBOHB, b. AtRBOHD, c. AtP5CS1, d. AtNHX7, e. AtSOD, f. AtCAT, g. AtPOD, h. AtRD22, i. AtRD29A, j. AtRD29B, k. AtNAC3, l. AtWRKY46, m. AtERF, n. AtMYB5. Mock, without salt treatment, NaCl: 200 mM NaCl treatment. *, p < 0.05 and **, p < 0.01 by Student’s t-test compared with untreated (Mock). Data represent means ± SD. (TIF 1397 kb) [file 12870_2019_2088_MOESM5_ESM.tif]
